# Developmental transitions: integrating environmental cues with hormonal signaling in the chromatin landscape in plants

**DOI:** 10.1186/s13059-017-1228-9

**Published:** 2017-05-10

**Authors:** Jun Xiao, Run Jin, Doris Wagner

**Affiliations:** Department of Biology, University of Pennsylvania, Philadelphia, PA 19104 USA

## Abstract

Plant development is predominantly postembryonic and tuned in to respond to environmental cues. All living plant cells can be triggered to de-differentiate, assume different cell identities, or form a new organism. This developmental plasticity is thought to be an adaptation to the sessile lifestyle of plants. Recent discoveries have advanced our understanding of the orchestration of plant developmental switches by transcriptional master regulators, chromatin state changes, and hormone response pathways. Here, we review these recent advances with emphasis on the earliest stages of plant development and on the switch from pluripotency to differentiation in different plant organ systems.

## Introduction

Recent studies in both animals and plants have revealed that the epigenome contributes to cell identity and function [1, 2]. The epigenome comprises alternative chromatin states that can impact gene activity; they are not accompanied by alterations in nucleotide sequence but can nevertheless be passed on to daughter cells. It is now clear that a number of attributes of the chromatin impact the accessibility of the genome for transcription, including: the three-dimensional organization of the chromatin in the nucleus; chromatin condensation by linker histones and non-histone proteins; histone modifications or the presence of alternative histones (i.e., histone variants); the position and occupancy of the nucleosomes; and covalent modification of the DNA by methylation [3, 4]. Upon perceiving a relevant cue, enzyme complexes (Box 1) can alter the existing chromatin state, making new genomic regions accessible while closing others off, thus generating a “legible genome” that is specific to cell type, developmental stage, or environmental condition. In this review, we discuss some of the major developmentally or environmentally triggered transcriptional reprogramming events in plants, with special emphasis on the role of chromatin and the epigenome.

## Early stages in plant development and response to environmental cues

### From fertilization to embryo development

**Fig. 1:**
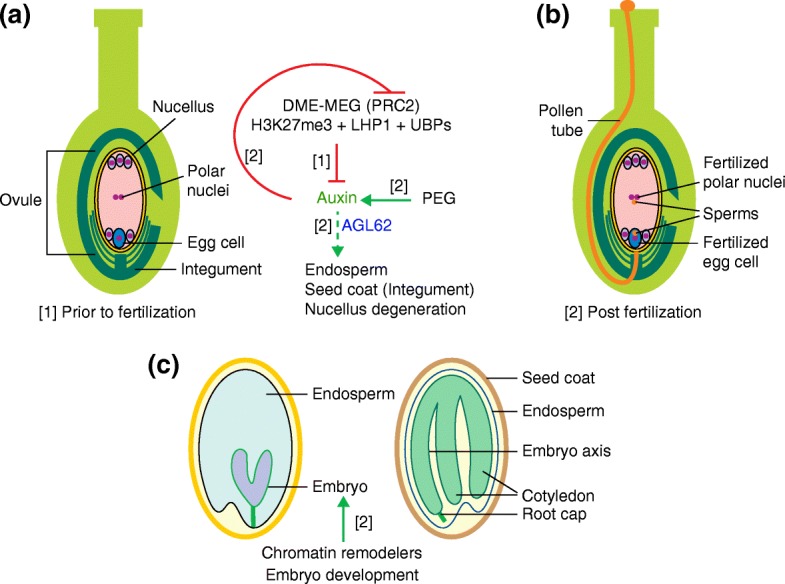
Function of chromatin regulators in seed development. **a** Prior to fertilization, DNA hypomethylation by DME enables maternal expression of PRC2, which deposits H3K27me3 at auxin response genes to inhibit the development of non-embryonic tissues. PRC2 acts in concert with LHP1 and histone H2A deubiquitinases (UBPs). **b** Post fertilization, paternally expressed genes raise the auxin hormone level and activate non-embryonic tissue development; this requires the endosperm expressed TF AGL62. The increased auxin levels reduce PRC2 expression. **c** CHD and SWI/SNF chromatin remodelers contribute to embryo pattering after fertilization. *Black*, chromatin regulators; *blue*, transcription factors; *green*, hormones

In angiosperms, seed development is initiated by a double-fertilization event, during which the egg cell and the central cell each fuse with a male sperm cell, resulting in the formation of the embryo and the endosperm, respectively (Fig. 1). The embryo and the endosperm are surrounded by maternal tissues such as the seed coat, which derives from the integuments [5]. Proper seed formation is achieved by the coordinated development of these three different tissue types [6]. The embryo initiates a shoot and a root apical meristem, two leaf-like structures called cotyledons, and a short stem termed the hypocotyl [7].

The endosperm is a nourishing tissue that supports embryo growth [8]. Its initiation and its correct development are necessary for the establishment of a viable seed [9]. Endosperm development prior to fertilization is inhibited by FIS (FERTILIZATION-INDEPENDENT SEED)-PRC2 (POLYCOMB REPRESSIVE COMPLEX2), which acts in the female gametophyte and during endosperm development. Loss of FIS-PRC2 function causes autonomous endosperm development without fertilization, resulting in seeds that develop an endosperm but no embryo [10]. More recently, histone ubiquitination and the hormone auxin were linked to endosperm formation. Two H2A deubiquitinases, Ubiquitin-Specific Protease 12 (UBP12) and UBP13, are partners of the H3K27me3-binding protein Like Heterochromatin Protein 1 (LHP1) [11]. These proteins are expressed in the central cell of the mature female gametophyte and are recruited to several Polycomb targets, where they are required for elevated H3K27me3 levels and for the repression of transcription. In the absence of LHP1 or UBP12/UBP13, autonomous endosperm develops, suggesting that LHP1 or UBP12/UBP13 may repress FIS-PRC2 targets [12] (Fig. 1a). Elevating auxin levels, either genetically or pharmacologically, induces replication of the central cell in the absence of fertilization [13], suggesting that auxin may promote endosperm formation. Indeed, FIS-PRC2 directly silences two auxin biosynthesis pathway genes, *YUCCA10* (*YUC10*) and *TRYPTOPHAN AMINOTRANSFERASE RELATED 1* (*TAR1*) in the maternal gametophyte; this lowers auxin levels in the central cell prior to fertilization. After fertilization, paternal expression of auxin biosynthesis genes enables an auxin increase in the fertilized central cell, which triggers the initiation of endosperm formation, bypassing the block by the maternal FIS-PRC2 [13] (Fig. 1b).

The endosperm is also the main site of genomic imprinting in flowering plants, an epigenetic phenomenon that results in the expression of a gene from just one of the two available alleles in a parent-of-origin-dependent manner [14]. Imprinting has evolved independently in mammals and flowering plants [15]. Differential DNA methylation underlies most imprinted gene expression [16]. Global removal of methylation from cytosines found in CG dinucleotides by the DEMETER (DME) DNA glycosylase occurs in the maternal genome of the endosperm in *Arabidopsis thaliana*, leading to hypomethylation [17, 18]. DME is expressed in the companion cells of the gametes, including the central cell of the female gametophyte before fertilization [19] (Fig. 1a, b). Genes that are exclusively maternally expressed (MEGs) are characterized by loss of repressive DNA methylation. Paternally expressed genes (PEGs) arise when reduced maternal DNA methylation enables an alternative epigenetic silencing mechanism—polycomb repression—to silence the maternal alleles [20, 21]. In some cases, parent-of-origin-specific H3K27me3 is not dependent on differential DNA methylation [22].

Among the numerous MEGs that have been identified are the FIS-PRC2 components MEDEA (MEA) and FIS2 [22]. As discussed above, mutation of the MEA or FIS2 components of FIS-PRC2 causes the formation of endosperm prior to fertilization and embryo abortion. This is due, at least in part, to de-repression of the maternal alleles of the PEGs *YUC10* and *TAR1* and to increased auxin levels [13, 23]. Interestingly, while some of the same genes (including the auxin biosynthesis genes) are imprinted in many different flowering plants, the majority of the imprinted genes are species-specific [24–26]. Moreover, imprinting at the same gene may be achieved by different mechanisms in different plant species. In *Arabidopsis lyrata*, an outcrossing plant species closely related to *A. thaliana*, many PEGs arise due to CHG methylation and repression of the maternal alleles, and the maternal endosperm genome is not hypomethylated [27]. One biological role of gene dosage or of imprinting in the endosperm may be as a hybridization barrier that underlies speciation [28, 29]. The maternal FIS-PRC2 may also buffer paternal genetic variation to prevent its influence on seed development [30]. Finally, imprinted gene expression may transmit environmental cues that are perceived by the mother plant to modulate seed germination [31].

A clever genetic trick has been used to enable egg cell fertilization in a *prc2* null mutant background [32]. This gave rise to viable embryos that became abnormal only after germination, pinpointing the developmental window during which PRC2 function is first required in plant development [33]. Thus, unlike in animals [34], PRC2 is not strictly essential for embryo formation in plants. Other chromatin regulators are important for the development of the embryo proper (Fig. 1c); for example, double mutants in the redundantly acting SWI/SNF (SWItch/Sucrose Non-Fermentable) subfamily chromatin remodelers MINUSCULE1 (MINU1) and MINU2 cause embryo lethality, with abnormal cell divisions apparent by the globular stage [35]. Double mutants in the BRAHMA (BRM) and SPLAYED (SYD) SWI/SNF subfamily chromatin remodelers, which have overlapping roles, also cause embryo lethality, as do mutations in the SWI/SNF chromatin remodeling complex components SWI3A or SWI3B [36–40]. In the case of *brm* mutants, the embryo defect may result from reduced auxin response; double mutants in *brm* and the auxin response factor *monopteros* (*mp*) are embryo lethal [40].

Unlike the egg cell and the central cell, which are fertilized and give rise to the embryo and the endosperm, the maternal tissue of the ovule does not participate in the fertilization process, yet it also undergoes drastic changes in response to fertilization. The integuments undergo rapid cell division and expansion to form the seed coat [41], while the proximal region of the nucellus undergoes programmed cell death (PCD) [42]. Sporophyte PRC2 (EMBRYONIC FLOWER2 (EMF2)/VERNALIZATION2 (VRN2)-PRC2) exerts a block on seed coat development before fertilization, and lack of the core PRC2 subunits VRN2 and EMF2 results in dosage-dependent autonomous seed coat development [43]. Auxin and gibberellin (GA) signaling are activated in the seed coat post-fertilization, and exogenous application of GA3 or 2,4-D (auxin) or overproduction of these hormones promotes fertilization-independent seed coat development [23]. The production of auxin in the unfertilized central cell is sufficient to drive seed coat development [23, 43], and the endosperm-specific transcription factor (TF) AGAMOUS-like MADS box protein 62 (AGL62) [44] promotes the transport of auxin from the endosperm to the integuments via the transcriptional upregulation of a PGP-type auxin transporter [23, 45]. Genetically, auxin and PRC2 act in the same pathway, with auxin acting upstream of PRC2 and downregulating PRC2 accumulation, whereas GA is activated when PRC2 is removed from the integuments (Fig. 1a, b). These findings uncover a precisely tuned developmental switch, operating at the intersection of hormones and chromatin regulators, that provides coordinated development of the embryo, endosperm, and seed coat. It also balances the maternal and paternal genomes, thereby impacting survival and speciation.

### Seed maturation and dormancy

**Fig. 2:**
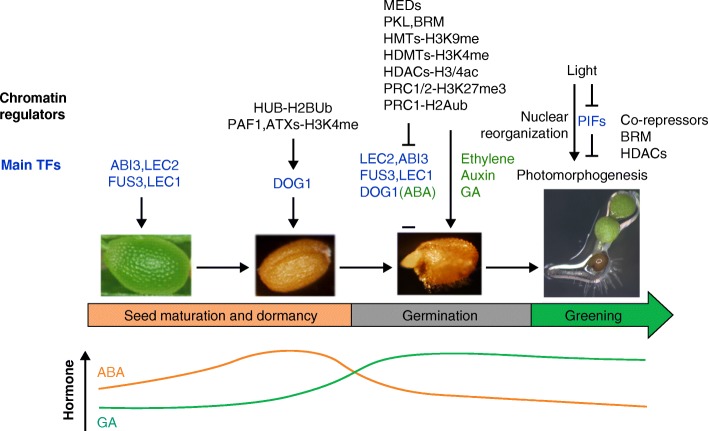
Involvement of chromatin regulators during seed maturation, dormancy, and germination. Master transcription factors (TFs), such as ABI3, LEC2, FUS3, LEC1, and DOG1, promote seed maturation and dormancy and trigger ABA hormone accumulation. The H2B ubiquitinase HUB, the PAF1 complex, and H3K4 methyltransferases (ATXs) promote *DOG1* transcription. Subsequently, during germination, a large number of different chromatin regulators jointly repress expression of the *LEC2*, *ABI3*, *FUS3*, *LEC1*, and *DOG1* TF and increase the GA/ABA hormone ratio. These chromatin regulators include MEDIATOR (MED) components, H3K9 methyltransferases (HMTs), H3K4 demethylases (HDMTs), HDACs, and the PRC1 and PRC2 Polycomb complexes. Finally, light signaling promotes photomorphogenesis, activates the seedling program, and triggers large scale nuclear reorganization, which is mediated by the PIF TFs and chromatin regulators such as BRM and HDACs

In higher plants, seed development can be divided into two phases, morphogenesis (embryo and endosperm development) and maturation. Maturation ensures that the fully developed embryo accumulates sufficient storage compounds, while water content decreases and abscisic acid (ABA) levels increase. Finally, the seed acquires desiccation tolerance and enters a metabolically quiescent state [46]. The initiation of seed maturation is mainly controlled by three B3 domain TFs: LEAFY COTYLEDON2 (LEC2), ABSCISIC ACID (ABA) INSENTITIVE3 (ABI3) and FUSCA3 (FUS3). These factors work in concert with the CCAAT-box binding complex component LEC1 to regulate ABA, auxin, GA, and sugar responses [47]. They form a complex gene regulatory network that activates largely overlapping downstream genes that are involved in starch and lipid biosynthesis. They also regulate the biosynthesis and/or catabolism of the hormones ABA and GA to balance their ratio during seed dormancy and germination [47, 48] (Fig. 2).

Seed dormancy is initiated during early seed maturation and continues after the seed completes its development, but then is gradually overcome during dry storage (after-ripening). The plant hormone ABA and the DELAY OF GERMINATION 1 (DOG1) protein are essential regulators of seed dormancy. ABA is produced during seed maturation and is necessary for the induction of seed dormancy and for maintenance of the dormant state following seed shedding. Factors that modulate ABA levels or signal transduction alter the seed dormancy level [49, 50]. *DOG1*, which was isolated by quantitative trait locus analysis, has been identified as a major and “specific” regulator of seed dormancy in *A. thaliana* and other plants, and DOG1 levels and activity are intricately regulated [51–53]. This regulation includes positive autoregulation at sites that are subject to natural variation [54]. A study of dormancy cycling in the soil implicated seed bank factors involved in the dynamics of chromatin remodeling in changing dormancy status by altering the expression of key regulators such as *DOG1* [55]. The H2B ubiquitinases HISTONE MONOUBIQUITINATION 1 (HUB1) and HUB2 and the ARABIDOPSIS TRITHORAX-RELATED 7 (ATXR7) H3K4 methyltransferase promote seed dormancy by upregulating expression of *DOG1* and other genes, presumably by influencing their H2Bub and H3K4 methylation statuses [56–58]. By contrast, SIN3-LIKE (SNL) co-repressor proteins promote seed dormancy by preventing the acetylation of H3K9/18 or H3K14 at genes linked to germination [59]. In addition, the HDA9 HDAC promotes seed dormancy by repressing genes that are related to photosynthesis and photoautotrophic growth [60, 61] (Fig. 2). During seed maturation, the expression of master transcriptional activators of seed dormancy is therefore upregulated by chromatin modifications that are potentially linked to transcriptional elongation, while genes that promote germination and photosynthesis are repressed by histone deacetylation.

### Seed germination and greening (photomorphogenesis)

After the release of dormancy by environmental signals such as stratification [62], seed germination commences with protrusion of the radicle through the seed coat [47]. This process is facilitated by GA, which is newly synthesized in the imbibed embryo, and is inhibited by ABA [47]. Additional hormones such as ethylene and auxin also play roles in germination [63]. Low doses of auxin promote germination, whereas high doses inhibit this process [63]. Upon germination, the seed maturation program is silenced and seedling identity genes are activated. The broad changes in transcriptional programs that accompany this key developmental transition are orchestrated by a myriad of events that remodel and modify chromatin state (Fig. 2).

Repression of the seed maturation/dormancy program involves both the EMF2-PRC2 complex and PRC1, which silence the expression of seed maturation loci such as *ABI3*, *LEC2*, *DOG1*, and *CHOTTO1* (*CHO1*)/*AINTEGUMENTA-LIKE 5* (*AIL5*) during germination [33, 64–69]. The PRC1 complex is recruited by VP1/ABI3-like (VAL) proteins and PHD domain-containing ALFIN1-like (AL) proteins. AL protein can bind to H3K4me3. Two homologs of ZUOTIN-RELATED FACTOR1 (ZRF1), possible readers of H2Aub, contribute to Polycomb-mediated silencing of *ABI3*, *CRUCIFERIN 3* (*CRU3*), and *CHO1*/*AIL5* [70]. Histone deacetylases (HDACs) such as HDA19 and HDA6 also repress seed maturation genes [71–74]; these enzymes are recruited by diverse TFs, including VAL2, SCARECROW-LIKE15 (SCL15), and BRI1-EMS-SUPPRESSOR1 (BES1), and by the TOPLESS (TPL) co-repressor [73, 74]. Finally, H3K9 methylation by SU(VAR)3-9 HOMOLOG 4 (SUVH4) and SUVH5 and chromatin remodeling by the chromodomain (CHD) family member PICKLE (PKL) and by the SWI/SNF chromatin remodeler BRM also contribute to the silencing of dormancy and of embryonic genes [75–79]. The histone H3K4me2/3 demethylases LYSINE SPECIFIC DEMETHYLASE LIKE 1 (LDL1) and LDL2, by contrast, assist in the process by removing activating histone modifications from the seed dormancy genes [80].

To promote germination, the histone arginine demethylases JUMONJI DOMAIN-CONTAINING PROTEIN 20 (JMJ20) and JMJ22 remove repressive histone arginine methylation from two GA biosynthesis genes, *GIBBERELLIN 3 BETA-HYDROXYLASE1* (*GA3OX1*) and *GA3OX2* [81]. In addition, SNL co-repressors slow the speed of seed germination by inhibiting auxin synthesis and directly repress the expression of auxin transporters such as AUXIN RESISTANT1 (AUX1) [82]. Increased H3 lysine 9 or 18 acetylation (H3K9/18 ac) at AUX1 was observed in *snl1 snl2* mutants. AUX1 enhances radicle emergence by promoting CYCLIN D expression [82].

When the seedling emerges from the soil, photomorphogenesis commences; this is characterized by reduced hypocotyl elongation, by cotyledon opening and expansion, and by chlorophyll biosynthesis [83]. The switch from heterotrophic to autotrophic growth is accompanied by large-scale transcriptional reprogramming in the context of chromatin (Fig. 2). Light exposure triggers nuclear architecture reorganization, which involves events such as nuclear size expansion, heterochromatin condensation and globally increased RNA Pol II activity [84]. This nuclear architectural change is induced mainly by blue light and is independent of local DNA methylation changes [84]. Germination is coupled to red/far-red light sensing by the light labile PHYTOCHROME-INTERACTING FACTORs (PIFs). For example, PIF1 inhibits seed germination in the dark by increasing ABA and by decreasing GA levels and response, as well as by repressing genes that are required for cell wall loosening [85]. PIF1 recruits the LEUNIG HOMOLOG (LUH) of the Groucho family transcriptional co-repressor to a subset of its targets [86]. PIF1 also inhibits chlorophyll biosynthesis by recruiting the SWI/SNF chromatin remodeling ATPase BRM to the chlorophyll biosynthesis gene *PROTOCHLOROPHYLLIDE OXIDOREDUCTASE C* (*PORC*) to repress its expression [87]. The CHD chromatin remodeling ATPase PKL is required for 80% of the gene expression changes triggered by GA [88]. Finally, CCAAT-box binding factors redundantly repress light-controlled hypocotyl elongation, interact with HDA15, and bind to the promoters of hypocotyl elongation genes such as *IAA10* and *XTH17* [89]. Germination and establishment of autotrophic seedling growth thus not only rely on chromatin modification and remodeling in response to environmental cues that trigger repression of the embryonic and dormancy programs, but are also accompanied by altered hormone environments and large scale nuclear reorganization.

## Pluripotency and differentiation in plant development

### De-differentiation and callus formation

**Fig. 3:**
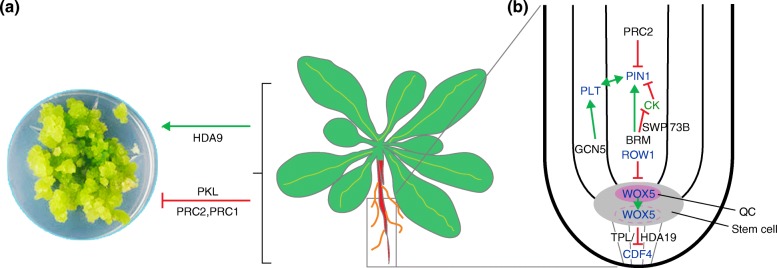
Role of chromatin remodelers in callus formation and root development. **a** Spontaneous de-differentiation of meristematic plant tissues is prevented by Polycomb repression (PRC2, PRC1), while the CHD chromatin remodeler PKL prevents induced callus formation. Histone deacetylation (HDA9) promotes de-differentiation in induced callus. **b** Stem cell maintenance is promoted by upregulation of auxin transport (via *PIN1*) through BRM/SWP73B and histone acetylation (GCN5) to promote expression of the *PLT* TFs. It also requires repression of *CDF4* by WOX5 and TPL/HDA19. Differentiation is promoted by PRC2 (which represses *PIN1* expression) and by ROW1, which prevents expansion of *WOX5* expression. *Black*, chromatin regulators; *blue*, transcription factors; *green*, hormones

All living plant cells can de-differentiate (i.e., form callus) when exposed to a combination of auxin and cytokinin (CK) hormones, and it has been proposed that de-differentiation occurs through a root developmental pathway [90]. Asexual propagation via induced de-differentiation and subsequent regeneration of a new plant is of economical importance for diverse species from oil palms to orchids [91]. Callus formation in plants—like induced pluripotency in animals —requires epigenetic reprogramming [92]. In agreement with these findings, callus formation is accompanied by rapid loss of H3K27me3 from many genes, including several that are linked to the auxin pathway [93]. However, induced differentiation from leaves also requires PRC2 activity, presumably to silence the foliar gene-expression program [93]. PKL opposes callus formation and mutants in which this chromatin remodeling ATPase is defective are hypersensitive to CK and show enhanced callus greening [94]. By contrast, several HDACs promote callus formation and are transcriptionally upregulated during callus induction [95]. For example, mutation of *HDA9* or *HD-TUINS PROTEIN 1* (*HDT1*) causes reduced callus formation that is correlated with a lack of meristematic gene activity (Fig. 3) [95].

A recent genome-wide transcriptome comparison between wild-type leaves and leaf explant-derived calli identified 10,405 differentially expressed genes [96]. Not surprisingly, key TFs involved in leaf development were downregulated in the calli. In addition, 115 genes that are involved in chromatin remodeling were differentially expressed in calli. Notably, the expression of chromatin regulators that act in opposition to Polycomb repression (H3K4 methyltransferases of the Trithorax family of proteins) was elevated; these chromatin regulators may promote the expression of meristematic genes (Fig. 3). Reprogramming of cell identity through de-differentiation is not perfect and frequently results in DNA hypomethylation [97]. In the case of the oil palm, hypomethylation of the retrotransposon *Karma* in the B class floral homeotic gene results in flower-patterning defects and failure to form seeds [97]. On the other hand meristematic cells in plants need to be protected from de-differentiation. Polycomb repression prevents spontaneous de-differentiation and the repressive marks set by PRC2 are crucial for maintaining the identity of differentiation programs [98]. In particular, loss of PRC2 function leads to loss of cell identity and to callus formation from meristems in the shoot and root [33, 98]. The spontaneous callus formed from meristematic tissues in polycomb mutants differs from the induced callus in that it frequently produces somatic embryos [33, 98, 99]. In conclusion, de-differentiation of mature plant tissues is accompanied by large-scale epigenetic reprogramming in response to hormonal cues; this can result in epigenome defects in asexually produced plants. At the same time, plant meristematic tissues require Polycomb repression to block spontaneous de-differentiation.

### Root formation and the root stem cell niche

Chromatin regulators have been implicated in the establishment and maintenance of the primary and lateral root meristems. The EMF2-PRC2 complex directly represses the expression of the auxin transport protein PIN-FORMED1 (PIN1), which is important for rootward auxin flux, and thus reduces auxin accumulation and meristematic activity in both the primary and lateral root [100]. As a consequence, the rate of lateral root initiation is increased in *prc2* mutants [100]. PRC2 is expressed in the meristem and in the vasculature, and upstream regulators that control the spatiotemporal accumulation of PRC2 at the transcriptional level have recently been identified [101]. By contrast, the SWI/SNF chromatin remodeler BRM directly activates the expression of *PIN1* in the root [102]. Knockdown of SWI/SNF Associated Protein 73 (SWP73) causes defective roots with short meristems that have increased CK levels [103]. SWP73 represses the expression of ATP/ADP isopentenyltransferase (IPT) enzymes that regulate the rate-limiting step in CK biosynthesis. SWP73 binds to the *IPT3* and *IPT7* loci and destabilizes a positive gene regulatory loop (Fig. 3) [103]. The histone acetyltransferase GENERAL CONTROL NONDEREPRESSIBLE 5 (GCN5) promotes expression of *PLETHORA* (*PLT*) genes, which act in a positive feedback with the auxin pathway to promote maintenance of the root stem cell niche [104]. Finally, deacetylation also plays a role in the root meristem. The *WUSCHEL HOMEOBOX5* (*WOX5*) gene is expressed in the quiescent center (QC) and promotes stem cell fate in the surrounding initial cells (Fig. 3b). WOX5 directly represses expression of the TF CYCLING DOF FACTOR 4 (CDF4), which promotes differentiation, in the QC and in the columella stem cells [105]. WOX5 protein moves into the columella stem cells and recruits the TPL/HDA19 repressor complex to lower H3 acetylation at the *CDF4* locus regulatory region. *WOX5* expression in turn is confined to the QC by the PHD domain-containing protein REPRESSOR OF WUSCHEL1 (ROW1) [106]. When ROW1 binds to the activating H3K4me3 marks on the *WOX5* promoter, it silences *WOX5* expression by an as yet uncharacterized mechanism, restricting shootward expansion of the *WOX5* expression domain (Fig. 3). The transition from cell proliferation to differentiation in the root is preceded by eviction of the canonical histone H3.1 and its replacement with the H3.3 histone variant [107]. Thus, a multilayered chromatin regulatory and hormonal network controls root meristem maintenance and size.

### SAM initiation and maintenance

**Fig. 4:**
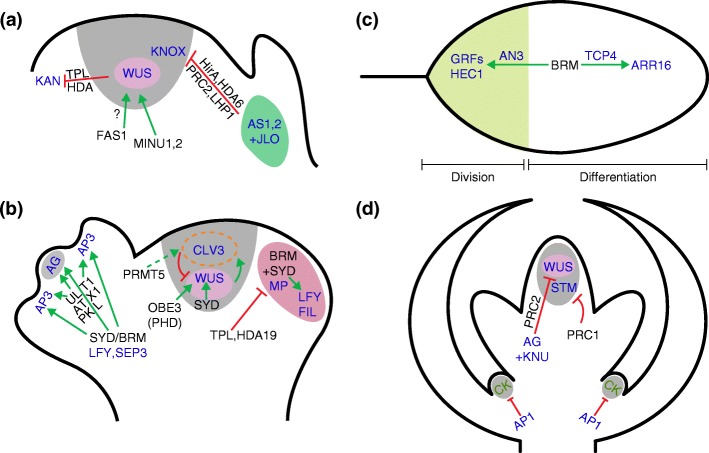
Chromatin remodelers regulate differentiation and proliferation. **a** In the organizing center of the shoot apical meristem, *WUS* expression is promoted by chromatin remodeling (via MINU1 and MINU2) and possibly chromatin assembly (involving FAS1). WUS in turn represses differentiation genes such as *KANADI1* (*KAN*) via histone deacetylation (involving TPL and HDA). In the leaf primordium, founder cell driver transcription factors (AS1, AS2, and JLO) repress *KNOX* gene expression via Polycomb repression, histone deacetylation, and histone variant incorporation (involving PRC2, LHP1, HDA6, and HirA). **b** In the inflorescence meristem center, stem cell maintenance is promoted by chromatin remodeling (SYD), histone arginine methylation (PRMT5), and a chromatin reader (OBE). SYD directly promotes expression of *WUS*, a positive regulator of stem cell fate. PRMT5, on the other hand, upregulates *CLV3*. CLV3 counteracts SAM overproliferation by inhibiting *WUS* expression. Flower primordium initiation at the flanks of the inflorescence meristem requires an auxin-triggered switch from a compacted chromatin state (TPL/HDA19) to an open chromatin state (BRM/SYD) at MP-bound target genes such as LFY and FIL that promote flower primordium fate. Finally, flower patterning requires the removal of Polycomb repression at floral homeotic genes such as *AP3* and *AG*. This is enabled by the concerted action of the chromatin remodelers SYD and BRM that are recruited by SEP3 and LFY. PKL and histone H3K4 methylation (ATX1, ULT1) also contribute to overcoming Polycomb repression at *AP3* and *AG*. **c** In different regions of the leaf, the chromatin remodeler BRM promotes either differentiation or cell division. Towards the tip of the leaf, BRM and TCP4 activate the *ARR16* TF gene. ARR16 inhibits CK response, which promotes differentiation. At the base of the leaf, BRM and AN3 induce expression of genes encoding the GRF and HEC1 TFs. GRF and HEC1 have been implicated in promoting cell proliferation in the leaf. **d** After floral organ initiation, the flower meristem terminates. The floral homeotic TF AG represses *WUS* expression directly and indirectly by promoting Polycomb repression. KNU, a direct target of AG, also represses WUS. The expression of *STM*, a KNOX gene, is silenced by PRC1. The class A floral homeotic gene AP1 lowers CK hormone levels to promote differentiation. *Black*, chromatin regulators; *blue*, transcription factors; *green*, hormones

Maintenance of the shoot apical meristem (SAM) is tightly controlled by opposite-acting pathways. WUSCHEL (WUS) and CLAVATA3 (CLV3) are two key stem cell regulators, with *WUS* being expressed specifically in the organizing center (OC) located below the stem cell pool (Fig. 4c). WUS non-cell-autonomously maintains stem cell identity by upregulating *CLV3* expression [108]. CLV3 is processed into a small peptide that limits *WUS* expression and prevents uncontrolled SAM proliferation [109]. Recently, the bHLH TF HECATE1 (HEC1) was shown to repress *WUS* and *CLV3* expression by integrating CK and auxin signals [110]. The TF FAR-RED ELONGATED HYPOCOTYL3 (FHY3) acts as a direct repressor of *CLV3*, thus maintaining the stem cell pool [111]. The GRAS family TF HAIRY MERISTEM (HAM) physically interacts with WUS/WOX proteins in various stem cell niches, and HAM and WUS regulate similar sets of genes [112]. *WUS* also represses the expression of the differentiation-related gene *KANADI1* (*KAN1*; Fig. 4a) [113]. Recently, the interaction between TPL/HDAC and WUS, which is required for stem cell fate promotion, was mapped to the WUS box and not to the EAR motif frequently implicated in transcriptional repression [114]. A separate pathway for SAM initiation and maintenance acts through the homeodomain TF SHOOTMERISTEM-LESS (STM), which induces CK biosynthesis [115]. CK acts as a positive regulator of *WUS* expression, mainly through the perception of CK by two CK receptors, ARABIDOPSIS HIS KINASE 2 (AHK2) and AHK4 [116]. A long-distance SAM-promoting pathway that involves the *bypass* (*bps*) signal has recently been uncovered [117]. *bps1* mutants fail to maintain meristem identity and *WUS* expression because of defects in CK response.

Only a handful of chromatin regulators have been implicated in SAM establishment and maintenance. FASCIATA1 (FAS1), a subunit of the *A. thaliana* homolog of CHROMATIN ASSEMBLY FACTOR-1 (CAF-1), which is responsible for replication-coupled incorporation of the canonical histone H3.1, is linked to stem cell maintenance [118]. *fas1* mutants enhance the SAM defects of weak *wus* mutants, yet *fas* single mutants have an expanded *WUS*-expressing domain and an enlarged SAM [118]. One explanation for this apparent discrepancy may be that *fas1* mutants fail to initiate a functional organizing center upon germination; this defect may result in the establishment of new *WUS*-expressing cell populations that subsequently fuse to form one large fasciated meristem (Fig. 4a). A similar sequence of events was observed in double mutants of the redundantly acting MINU1 and MINU2 SWI/SNF chromatin remodeling ATPases; hypomorph *minu1 minu2* mutants form multiple primary meristems [35]. PROTEIN ARGININE METHYLTRANSFERASE 5 (PRMT5), a member of the type II arginine methyltransferase family, directly binds to the regulatory regions of the gene encoding the CLV3 peptide receptor CORYNE (CRN) (Fig. 4b). The resulting H3R4me2 methylation represses *CRN* expression and this promotes meristem homeostasis [119]. Similarly, *OBERON3* (*OBE3*), which encodes a PHD finger-containing protein, acts as a positive regulator of *WUS* expression in a mutual positive feedback loop (Fig. 4b) [120]. Finally, the SWI/SNF chromatin remodeler SYD directly promotes the maintenance of *WUS* expression [121].

### Leaf development

To enable leaf initiation at the flanks of the shoot apex, the MYB TF ASYMMETRIC LEAVES 1 (AS1) and its partner the LATERAL ORGAN BOUNDARY (LBD) domain TF AS2 repress the expression of pluripotency genes. AS1 and AS2 directly recruit the HirA histone H3.3 chaperone and PRC2 to the regulatory regions of the Class-I *KNOTTED1-like homeobox* (*KNOX*) family genes *BREVIPEDICELLUS* (*BP*) and *KNOTTED-LIKE FROM ARABIDOPSIS THALIANA 2* (*KNAT2*) to silence them [122, 123]. The LBD protein JAGGED LATERAL ORGAN (JLO) contributes to AS2-mediated *KNOX* repression by forming a trimeric complex with AS1. Loss of JLO function leads to the ectopic expression of *STM* and *BP* [124]. The HDAC HDA6 also interacts with AS1 and directly represses *KNOX* gene expression [125]. More recently, LHP1, also known as TERMINAL FLOWER2 (TFL2), was shown to contribute to *KNOX* gene repression via direct physical interaction with AS1 and AS2 (Fig. 4a) [126]. LHP1 may promote the spread of H3K27me3 [127]. LHP1 and AS1/AS2 have many additional direct targets that have roles in leaf development and maturation [126, 128].

*A. thaliana* leaf cells also face a choice between proliferation and differentiation. Leaf differentiation is promoted by recruitment of the chromatin remodeler BRM and the dedicated BRM complex component SWI3C by the CINCINNATA-like TEOSINTE BRANCHED1, CYCLOIDEA, and PCF (CIN-TCP) TF TCP4 [129]. BRM, together with TCP4, reduces CK responsiveness by promoting the expression of an inhibitor of CK response, *ARABIDOPSIS RESPONSE REGULATOR 16* (*ARR16*). The transcription co-activator ANGUSTIFOLIA3 (AN3), on the other hand, promotes cell proliferation in leaves [130]. AN3 directly induces the expression of *GROWTH REGULATING FACTORS* (*GRFs*) and *HEC1*. These genes are also direct targets of the SWI/SNF complex components SWP73B and BRM, with which AN3 physically interacts (Fig. 4c). A subsequent study additionally implicated SWP73B in leaf polarity [131]. Similar interactions and roles for AN3 and SWI/SNF were also observed in maize leaf development [132]. Thus, AS1 is at the center of a chromatin repressor hub that promotes leaf initiation, whereas opposite roles of the SWI/SNF complex in leaf maturation are distinguished by the presence of the AN3 co-activator (Fig. 4b, c).

### Flower development

Organogenesis (flower primordium initiation) from stem cell descendants at the periphery of the inflorescence meristem requires an auxin maximum that activates the AUXIN RESPONSE FACTOR 5 (ARF5) or MONOPTEROS (MP) [133]. When auxin levels are low, negative regulators of auxin response, the auxin-labile AUX/IAA proteins, bind to ARFs such as MP and generate a repressive chromatin environment [133]. AUX/IAA proteins physically interact with and recruit the TPL/HDA19 co-repressor and additionally prevent MP from interacting with the SWI/SNF ATPases SYD and BRM [40, 134]. The histone deacetylation generates a repressive chromatin environment near MP-binding sites that prevents the activation of auxin response genes in the absence of the hormonal cue. Increased auxin levels in the primordium founder cells lead to AUX/IAA protein degradation, loss of TPL/HDA19, and physical interaction of SWI/SNF complexes with MP. SYD and BRM open up the chromatin at MP target loci such as *LEAFY* (*LFY*) and *FILAMENTOUS FLOWERS* (*FIL*) [40]. Histone acetylation might also contribute to this process. For example, the bZIP11 TF recruits the GCN5 histone acetyltransferase to promote the expression of auxin biosynthesis genes [135].

Floral meristems (FMs) arise from subapical stem cells in the center of the inflorescence [136] and give rise to the primordia of the floral organs [137]. In stage 2 flowers, the FM is fully formed and floral organ primordium patterning is initiated by the activation of the floral homeotic genes. Prior to this developmental time point, floral homeotic genes are silenced by Polycomb repression [137]. ARABIDOPSIS TRITHORAX 1 (ATX1) promotes upregulation of the floral homeotic genes through H3K4 tri-methylation [138]. In addition, the master regulator of floral cell fate, LFY, together with the MADS-domain TF SEPALLATA3 (SEP3) recruits the SWI/SNF chromatin remodelers BRM and SYD to the class B and class C floral homeotic genes [39]. The activity of the remodelers is absolutely required to upregulate the floral homeotic genes, and the combined LFY and SEP3 expression domains in the FM overlap with the sites where these floral homeotic genes are induced. The SAND domain-containing protein ULTRAPETALA1 (ULT1) acts in parallel with LFY to activate the C class floral homeotic gene *AGAMOUS* (*AG*) [139]. Finally, the CHD chromatin remodeler PKL also promotes flower patterning and upregulation of the floral homeotic genes [140].

Interestingly, flower patterning and the activation of the floral homeotic genes is linked to flower meristem termination [137]. Like the vegetative SAM and the reproductive inflorescence meristem, FMs express the pluripotency factors *WUS* and *STM* [135] (Fig. 4d). The class C floral homeotic gene *AG* directly represses the expression of the stem cell-promoting gene *WUS* in the center of the flower meristem with the help of PRC2 [141]. In addition, AG activates the zinc finger protein KNUCKLES (KNU), which in turn directly represses *WUS* and thus terminates meristem identity (Fig. 4d) [142]. ARF3 binds to the chromatin of *WUS* in an AG-dependent manner and directly represses *WUS* expression to promote FM determinacy [143]. In addition, the SAND domain protein ULT1 represses *WUS* expression, working together with its partner ULT1 INTERACTING FACTOR 1 (UIF1), a MYB and EAR domain-containing TF that can bind to *WUS* regulatory regions [144]. In parallel, AtRING1a and AtRING1b (core components of the PRC1 complex) contribute to the termination of floral stem cell fate through repression of *KNOX* genes [145]. Finally, the class A floral homeotic gene *AP1* suppresses meristematic activity in the axils of the outermost floral organs, the sepals, by lowering CK levels (Fig. 4d). AP1 directly represses the expression of the CK biosynthetic gene *LONELY GUY1* (*LOG1*) and directly upregulates the CK degradation gene *CYTOKININ OXIDASE*/*DEHYDROGENASE3* (*CKX3*) (Fig. 4d) [146]. AP1 can physically interact with transcriptional co-repressors linked to histone deactylation and with SWI/SNF group chromatin remodelers [147–149]. Thus, in flowers, tightly regulated chromatin state switches promote organ initiation, flower patterning, and meristem termination.

## Discussion

The picture that emerges from the recent investigations is that developmental transitions in plants are orchestrated by the combined activities of transcription factors, hormone response pathways, and regulators of chromatin state. There is crosstalk between these three regulatory layers. For example, transcription factors recruit chromatin enzymes but are also dependent on chromatin remodeling for the ability to bind target genes. The hormonal pathways trigger chromatin state changes, and chromatin modification and remodeling alter hormone accumulation, signaling, and response. Finally, hormone environments alter transcription factor activity and transcription factors modulate hormone levels and response. In addition, the large-scale transcriptional reprogramming that occurs during major developmental switches relies on many diverse chromatin regulators; this enhances both the robustness of the underlying chromatin state changes and the plant's ability to fine-tune the response to diverse cues. Other conclusions are less universal. For example, while Polycomb repressive complexes and SWI/SNF chromatin remodeling ATPases frequently act in opposition, they can also jointly promote a specific reprogramming event by acting on the same or on different targets.

A longstanding question has been whether the writers, erasers, and readers of the chromatin state changes that accompany major reprogramming events are permissive—working by allowing master transcriptional regulators to exert their roles in transcriptional reprogramming or by preventing them from doing so—or whether they can also be decisive, that is to say they can interpret intrinsic and extrinsic cues to trigger the reprogramming events. While the jury is still out on this question, what has become clear is that the boundaries between TFs and the chromatin regulators are becoming more and more blurred. Some TFs in plants are more promiscuous in their genome occupancy than chromatin regulators [150]. In addition, as outlined above, it has become apparent that many developmental or environmental cues are directly interpreted by chromatin regulators and modulate their spatial, temporal, and condition-dependent accumulation or activity [101, 151–154].

To better understand developmental transition in the context of chromatin in plants, the future presents a number of challenges:

1. To elucidate the cell-, tissue- and condition-dependent roles of chromatin regulators using spatially restricted loss- and gain-of-function mutants in these regulators combined with cell- and tissue-specific epigenome analyses.
2. To identify in temporal resolution the order and logic of the series of chromatin state changes that lead to the repression and activation of new gene expression programs.
3. To define the composition of the individual or multifunctional complexes that trigger chromatin state changes and to determine how their formation and activity are controlled by extrinsic or intrinsic cues.
4. To uncover the biological roles in plant development or stress responses of the large number of predicted chromatin regulators present in plant genomes whose biological roles are not yet understood.

## Box 1. Chromatin regulators

Genomic DNA that is wrapped around the histone octamer in nucleosomes is much less accessible than DNA that is not in contact with histones. Nucleosome occupancy (the fraction of a specific genomic DNA fragment that is wrapped around a histone octamer in a population of cells/nuclei) or nucleosome positioning (the identity of the specific DNA fragment wrapped around the histone octamer in a larger region of interest) can be altered by chromatin remodeling using the energy derived from ATP hydrolysis to break the histone–DNA contacts [155]. Plants have a large number of chromatin remodelers, but the SWItch/ Sucrose Non-Fermentable (SWI/SNF) complexes formed around BRAHMA (BRM) and SPLAYED (SYD) and the chromodomain (CHD) family chromatin remodeling ATPase PICKLE (PKL) are the most studied [156].

Histone variants are predominantly incorporated into nucleosomes outside of DNA replication and differ in primary sequence from “canonical” histones. These sequence differences impact the properties of the histone variants and those of the nucleosome particles that contain them [157]. In this review, the histone variants H2A.Z, H3.3, and H1.3 are discussed.

Covalent modification of histones is executed by “writers”—enzymes that covalently alter amino acids in the histones through acetylation, methylation, ubiquitylation, or phosphorylation, for example [158]. Many of these enzymes act in complexes. Histone acetyltransferases (HATs) generally cause increased genome accessibility (less compaction), whereas the effects of lysine methylation are strongly context dependent. Polycomb Repressive Complex 2 (PRC2) generates tri-methylation on lysine 27 of histone H3 (H3K27me3), a transcription-repressive mark, whereas tri-methylation on lysine 4 of histone H3 (H3K4me3) is associated with open chromatin and active transcription. Histone arginine methylation is frequently repressive. Ubiquitination on lysine 121 of histone H2A (H2AK121ub) is generated by PRC1, an enzyme complex that also contains non-histone proteins that strongly compact chromatin. Ubiquitination on lysine 143 of histone H2B (H2BK143ub) promotes transcriptional elongation.

Additional non-histone proteins have specific protein domains (such as PHD domains) that can recognize histone modifications; these downstream effectors are called “readers”. Readers interpret the chromatin state and contribute to the final chromatin compaction and transcription outcome.

Finally, all covalent histone marks are reversible, their removal being executed by so-called “erasers”. There are a myriad of erasers in plants; in this review, histone deacetylases (HDACs), which remove histone lysine acetylation, feature most prominently. HDACs, on their own or together with Polycomb repression, compact chromatin to silence unnecessary or detrimental gene expression programs.

In plants, cytosine DNA methylation occurs in three sequence contexts: CG, CHG, and CHH (where H equals A, T, or C) [159]. Specialized complexes have been linked to the initiation, maintenance, and removal of cytosine methylation. Cytosine methylation is frequently associated with transposable elements, and some of these transposable elements have been co-opted for the transcriptional regulation of nearby genes, generally silencing gene expression when methylated. Removal of CGme is executed by the DEMETER (DME) DNA glycosylase, which has prominent roles in imprinting in the endosperm.

## Abbreviations

ABA: Abscisic acid
ABI3: ABA INSENTITIVE3
*AG*: *AGAMOUS*
AHK2: ARABIDOPSIS HIS KINASE 2
*AIL5*: *AINTEGUMENTA-LIKE 5*
AN3: ANGUSTIFOLIA3
ARF5: AUXIN RESPONSE FACTOR 5
AS1: ASYMMETRIC LEAVES 1
ATX1: ARABIDOPSIS TRITHORAX 1
AUX1: AUXIN RESISTANT1
*BP*: *BREVIPEDICELLUS*
*bps*: *bypass*
BRM: BRAHMA
CDF4: CYCLING DOF FACTOR 4
CHD: Chromodomain
*CHO1*: *CHOTTO1*
CK: Cytokinin
CLV3: CLAVATA3
CRN: CORYNE
DME: DEMETER
DOG1: DELAY OF GERMINATION 1
EMF2: EMBRYONIC FLOWER2
FAS1: FASCIATA1
FIS: FERTILIZATION-INDEPENDENT SEED
FM: Floral meristem
GA: Gibberellin
*GA3OX1*: *GIBBERELLIN 3 BETA-HYDROXYLASE1*
GCN5: GENERAL CONTROL NONDEREPRESSIBLE 5
HAM: HAIRY MERISTEM
HDAC: Histone deacetylase
HEC1: HECATE1
HUB1: HISTONE MONOUBIQUITINATION 1
IPT: Isopentenyltransferase
JLO: JAGGED LATERAL ORGAN
JMJ20: JUMONJI DOMAIN-CONTAINING PROTEIN 20
KNOX: KNOTTED1-like homeobox
KNU: KNUCKLES
LBD: LATERAL ORGAN BOUNDARY
LDL1: LYSINE SPECIFIC DEMETHYLASE LIKE 1
LEC2: LEAFY COTYLEDON2
*LFY*: *LEAFY*
LHP1: Like Heterochromatin Protein 1
MEA: MEDEA
MEG: Maternally expressed gene
MINU1: MINUSCULE1
*mp*: *monopteros*
PEG: Paternally expressed gene
PIF: PHYTOCHROME-INTERACTING FACTOR
PIN1: PIN-FORMED1
PRC2: POLYCOMB REPRESSIVE COMPLEX2
QC: Quiescent center
ROW1: REPRESSOR OF WUSCHEL1
SAM: Shoot apical meristem
SEP3: SEPALLATA3
SNL: SIN3-LIKE
STM: SHOOTMERISTEM-LESS
SUVH4: SU(VAR)3-9 HOMOLOG 4
SWI/SNF complex: SWItch/Sucrose Non-Fermentable chromatin remodeling complex
SWP73: SWI/SNF Associated Protein 73
SYD: SPLAYED
*TAR1*: *TRYPTOPHAN AMINOTRANSFERASE RELATED 1*
TF: Transcription factor
TPL: TOPLESS
UBP12: Ubiquitin-Specific Protease 12
ULT1: ULTRAPETALA1
VAL: VP1/ABI3-like
VRN2: VERNALIZATION2
*WOX5*: *WUSCHEL HOMEOBOX5*
WUS: WUSCHEL
*YUC10*: *YUCCA10*
